# Contribution of Chronic Sleep Deprivation to Age-Related Neurodegeneration in a Mouse Model of Familial Alzheimer’s Disease (5xFAD)

**DOI:** 10.3390/neurolint15030049

**Published:** 2023-06-27

**Authors:** Maria O. Klimenko, Tatiana A. Mishchenko, Yaroslava I. Mitaeva, Elena V. Kondakova, Elena V. Mitroshina, Maria V. Vedunova

**Affiliations:** Institute of Biology and Biomedicine, Lobachevsky State University of Nizhny Novgorod, 23 Gagarin Ave., 603022 Nizhny Novgorod, Russia

**Keywords:** sleep deprivation, aging, neurodegeneration, Alzheimer’s disease, 5xFAD mice, behavior, memory

## Abstract

Sleep–wake cycle disorders most often accompany the elderly and are frequently associated with the development of neurodegenerative processes, primarily Alzheimer’s disease. Sleep disturbances can be diagnosed in patients with AD even before the onset of memory and cognitive impairment, and become more pronounced as the disease progresses. Therefore, the expansion of our knowledge of how sleep relates to AD pathogenesis needs to be addressed as soon as possible. Here, we investigated the influence of chronic sleep deprivation on the motor and orienting–exploratory activity of 5xFAD mice, as well as their spatial learning ability and long-term memory retention. The studies carried out revealed that chronic sleep deprivation negatively affects the processes of spatial memory reconsolidation in 5xFAD mice. This leads to the development of stress-related behavioral responses, including aggressive behavior. In addition, the morphological changes in the cerebral cortex, including changes in the nuclear–cytoplasmic ratio and degradation of neuronal processes are observed. Moreover, we found an increase in the level of total DNA methylation in the blood of the sleep-deprived mice, which may be one of the mechanisms of the two-way relationship between sleep and neurodegeneration.

## 1. Introduction

Sleep disorders are a broad group of conditions encompassing all types of sleep-related disorders that affect a wide range of aspects of global health. There is a plethora of causes of sleep–wake cycle disruptions, including a gradual increase in anthropogenic load, stress, depression, changes in lifestyle and dietary habits, diseases that cause pain, difficulty breathing or frequent urination, etc. [1,2]. It has been found that women are more prone to sleep disorders than men [1]. Moreover, since the incidence of sleep disturbances becomes more frequent with age, the correlation between the severity of their manifestations and the quality of life of the elderly becomes particularly relevant. Nearly half of adults aged 60 years and older suffer from sleep disorders [3]. In addition to deterioration in general physiological and psycho-emotional status, the development of daytime fatigue, and a decreased ability to perform daily tasks, sleep disorders contribute to cognitive decline (i.e., memory, learning, logical reasoning, and mathematical operations) [4].

Chronic insomnia is one of the most severe forms of sleep disturbance, which is closely linked to age-related changes in the body, including brain functioning. It has been shown that changes in brain plasticity with aging are highly correlated with the restructuring of the morpho-functional architecture of neuron–glial networks [5], accompanied by increased cortical signaling in response to sleep–wake cycle disturbances [6,7]. Activation of microglia and an increase in their quantity at the site of presynaptic terminals in the chronic sleep deprivation state have been reported in different age groups [8]. We have previously demonstrated that young and adult female mice respond differently to the effects of long-term sleep deprivation [9]. In a normal state, adult female C57BL/6 mice (7–9 months) were less receptive to spatial learning than young mice (1.5 months). Mnestic functions in young mice were activated following chronic sleep–wake cycle disturbance, whereas severe memory impairment and reduced adaptive capacity of brain cells, including increased level of age-related PLIN2 protein expression and a downward tendency in DNA methylation levels, were observed in adult mice [9]. Our findings suggest that sleep deprivation increases the risk of pathological reactions and neurodegenerative processes with age.

However, the contribution of insomnia to age-related changes in the brain and its ability to activate neurodegeneration needs to be further investigated. Particular attention is given to the ability of sleep disorders to increase the risk of amyloid beta deposition leading to the development of Alzheimer’s disease (AD) [7]. AD is the most common type of dementia [10], affecting more than 50 million people worldwide, according to the World Health Organization [11]. On the one hand, sleep deprivation is considered to be a consequence of AD [12]. On the other hand, several studies have shown that sleep–wake cycle disturbances can provoke neurodegeneration in AD and may also cause an early manifestation of the disease [7]. One of the proposed mechanisms by which insomnia may trigger the development of AD is through decreased clearance of amyloid beta by astrocytes, aggregation of tau proteins, decreased production of the brain-derived neurotrophic factor (BDNF), and the development of inflammatory responses [8,10,13]. Since AD has very diverse phenotypic manifestations and progression, the role of sleep deprivation in pathological aspects of AD development and the features of neurodegenerative processes in the context of chronic insomnia have not been practically investigated. This is an area of growing interest given the fundamental differences in the brain’s ability to adapt to chronic sleep deprivation in different age groups. In the present study, using 5xFAD transgenic female mice, we performed a comprehensive analysis of the role of chronic sleep deprivation in the pathogenesis and progression of a familial form of AD in different age groups.

## 2. Materials and Methods

### 2.1. Research Object

The studies were performed in female 5xFAD mice and their non-transgenic littermate controls (WT) of two age groups—7–9 months and 10–12 months. The use of 5xFAD transgenic mice allows the modeling of a familial form of AD characterized by the formation of amyloid plaques in the brain [14,15]. The 5xFAD mouse line overexpress human APP695 and PSEN1 genes under the control of the mouse Thy1 promoter. The mutant human APP695 carries the Swedish mutation (K670N, M671L), the London mutation (V717I), and the Florida mutation (I716V), whereas human PSEN1 harbors two FAD mutations (M146L and L286V) [16].

Mice were divided into the groups described in Table 1.

The mice were housed in a certified SPF vivarium of Lobachevsky State University. The total number of animals was 170. Thirty mice were used for optimization of chronic sleep deprivation model regimens for the 5xFAD line. 

### 2.2. Chronic Sleep Deprivation Model

**Figure 1:**
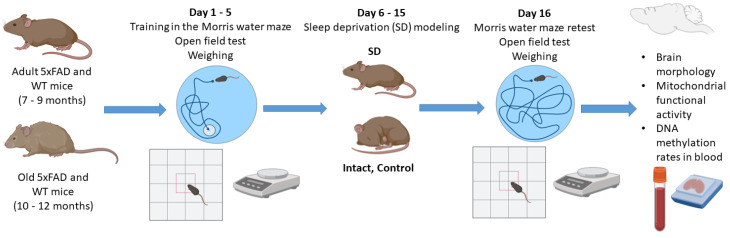
Scheme of the experiment.

Female mice were subjected to a flowerpot method developed by M. Jouvet and colleagues to simulate the deprivation of the rapid eye movement (REM) stage of sleep, with some modifications [9,17]. Mice were placed on a platform (d = 3 cm) located 1–2 cm below the water level of the circular pool at 10–11 a.m. and kept awake for 9 h a day for 10 days. The duration of sleep deprivation was chosen based on preliminary findings on the survival rate of mice aged 7–9 months, the mortality of which dramatically increased with more than 9 h of wakefulness. Five days after the start of sleep deprivation, the mice were additionally given an audiogenic signal every hour starting at 5 a.m. Body weight, as well as general motor and orienting–exploratory activity, was registered before and the day after the sleep deprivation modeling. In addition, spatial learning and memory in mice was assessed using the Morris water maze test. After the chronic sleep deprivation, the long-term memory retention test was performed, and the mouse brain samples were collected for further assessment of mitochondrial functional activity and histological analysis. Blood samples were obtained for analysis of DNA methylation rates using a DNA comet assay (Figure 1).

### 2.3. Open Field Test

The general motor and orienting–exploratory activity of mice was analyzed in an open field box (LE800S; Panlab Harvard Apparatus, Barcelona, Spain). Each mouse was placed in the setup and its behavior was recorded for 5 min using a Sony SSC-G118 camera (Tokio, Japan). The behavioral reactions registered by the mice were then evaluated using Smart 3.0.03 software (Panlab Harvard Apparatus, Barcelona, Spain), including an orienting–exploratory activity (the number of squares crossed around the perimeter of the arena, the number of upright postures, the number of peeks into holes), emotional state (the number of squares crossed in the center of the arena, the time spent in the center of the arena), and passive reaction anxiety (the number of grooming acts, acts of urination and defecation).

### 2.4. Morris Water Maze Test

Prior to sleep deprivation modeling, the mice were trained for 5 days in a circular pool (d = 90 cm) with a platform (d = 10 cm) placed 1–2 cm below the turbid warm water surface according to the previously developed protocol [9,18]. The duration of each training session was 60 s. The retest in the Morris water maze was performed the day after the sleep deprivation model. The mice were placed in the pool without a platform for 60 s. The time spent in the sector where the platform was previously located and the search strategy chosen by the mice to reach the target were assessed.

### 2.5. Brain Cell Mitochondrial Functional Activity Assessment

Brain mitochondria were isolated using standard differential centrifugation [9,19,20]. All procedures were performed on ice using ice-cold reagents. Each mouse was sacrificed by cervical vertebra dislocation, decapitated, and the brain was surgically isolated from the skull. The cerebellum was dissected, and the brain was placed in the porcelain mortar and homogenized in an isolation medium (210 mM mannitol, 70 mM saccharose, 10 mM HEPES, 0.1 mM EDTA (pH 7.4)). The obtained brain homogenate was centrifuged at the following regimens: 1100× *g* for 10 min at 0 °C (2 times) and 8500× *g* for 15 min at 0 °C. The isolated mitochondria were placed in an incubation medium containing 120 mM KCl, 5 mM NaH_2_PO_4_, 10 mM HEPES, 5 mM glutamate, 5 mM malate, and 14 mM MgCl_2_ (pH 7.4). Brain mitochondrial oxygen consumption was measured in the closed chamber of a high-resolution respirometer Oxygraph-2k (Oroboros Instruments GmbH, Innsbruck, Austria) at 37 °C with constant stirring. The Bradford method was used to determine the concentration of mitochondrial protein in a chamber (0.5 mg/mL).

The functional activity of brain mitochondria was assessed with the following parameters: V4—the activity of oxygen consumption by the mitochondria in the presence of respiratory chain complex I substrates (5 mM glutamate and 5 mM malate), V3—the rate of oxidative phosphorylation in V4 conditions in the presence of 5 mM adenosine diphosphate (ADP). The intensity of the mitochondrial respiratory chain complex II was assessed via sequential inhibition of complex I with rotenone (0.5 μM) and then stimulation of complex II with sodium succinate (10 mM).

### 2.6. Analysis of DNA Methylation Rates in Blood

The level of DNA methylation in mouse blood after chronic sleep deprivation modeling was assessed using a comet assay developed by J. Wentzel and colleagues, with some modifications [9,21]. Briefly, blood samples were mixed with 0.5% agarose and then placed on a glass slide pretreated with 1% agarose. The slides were stored overnight at 4 °C in a buffer containing 0.4 mM Na_4_EDTA, 2.5 M NaCl, 10% DMSO, and 1% Triton X-100 (pH 8). At the end of incubation, the slides were washed in a buffer containing 2 mM EDTA, 10 mM NaCl, 10 mM TrisHCl, and 1 mM B-mercaptoethanol (pH 7.9). Next, the samples were treated with HpaII or MspI enzyme mixture (15 U/mL, CutSmart Buffer, New England Biolabs, Ipswich, MA, USA), and incubated for 1 h at 37 °C in a humidity chamber precovered with a coverslip. 

After electrophoresis (1.0 V/cm, 300 mA, 4 °C for 45 min), the slides were fixed in ethanol followed by staining with VECTASHIELD^®^ Antifade Mounting Medium with DAPI (Vector Laboratories, Newark, NJ, USA). The resulting samples were examined in an LSM 800 confocal laser scanning microscope (Zeiss, Oberkochen, Germany). At least 100 cells per sample were counted, and the percentage of DNA in comet tails (Tail DNA, %) was determined. The percentage of CpG dinucleotide methylation was calculated using a CometScore 2.0 software.

### 2.7. Brain Morphology Assessment

Mouse brains were surgically removed from the skull and placed in a 10% formalin solution at room temperature for 24 h. Next, the brain samples were incubated in a 15% sucrose solution (24–48 h) and then stored in a 30% sucrose solution for 24–48 h. Each brain was then placed in a Leica CM1520 freezing sliding cryostat (Leica, Wetzlar, Germany), progressively filled with Cryogel (Leica, Wetzlar, Germany) and sliced into 10 µm coronal sections. Every fifth brain section was mounted on a glass slide, air-dried for 24 h, and stained with hematoxylin–eosin (PanReac AppliChem, Darmstadt, Germany). The brain sections were dehydrated in increasing concentrations of alcohol, purified in xylene, and embedded in mounting medium (Thermo Fisher Scientific, Waltham, MA, USA). The resulting samples were examined with a Zeiss Primo Star light microscope (Zeiss, Oberkochen, Germany) with an integrated Axio CamMRc camera (Zeiss, Oberkochen, Germany).

### 2.8. Statistical Analysis

The data were statistically processed using a GraphPad Prism v.9.3.1.471 software (San Diego, CA, USA). The normality of the results distribution was verified using the Shapiro–Wilk test. Differences between two independent groups were assessed by using the Mann–Whitney test. The Wilcoxon *t*-test was applied to determine the equality of the mean values in the two groups. The ANOVA test was used in the analysis of weight difference, data on the open field test, and mitochondrial functional activity. The data are presented as the mean ± standard error of the mean (SEM). Differences were considered to be statistically significant if the p value was less than 0.05.

## 3. Results

To assess the contribution of chronic sleep deprivation to the age-related development of neurodegenerative processes in the familial form of AD, we performed a comparative analysis of two age groups of female 5xFAD mice: 7–9 months–adult animals with pronounced signs of memory impairment, and 10–12 months–animals of the older age group with signs of aging (i.e., a loss of fur luster, graying of fur mainly on the back, loss of fur around the eyes and head up to 15–20%). Preliminary studies have shown a dramatic decrease in survival of 5xFAD mice deprived of sleep for 11 h per day (80% mortality rate). Therefore, the protocol of the sleep deprivation model was modified, and the time of forced activity was reduced to 9 h per day.

It is known that disturbances in a sleep–wake cycle cause disruption in melatonin metabolism resulting in altered feeding behavior and increased food intake [22,23]. Analysis of eating behavior showed a change in body weight of mice after chronic sleep deprivation modeling. The older 5xFAD group (10–12 months) showed a significant 3.8-fold increase in weight after sleep deprivation as compared to both the WT group within the 10–12 months age group (5xFAD 4.6 ± 2.5 g and WT −0.6 ± 0.5 g), and the 7–9 months SD group (5xFAD 0.7 ± 0.6 g) (Table 2).

**Figure 2:**
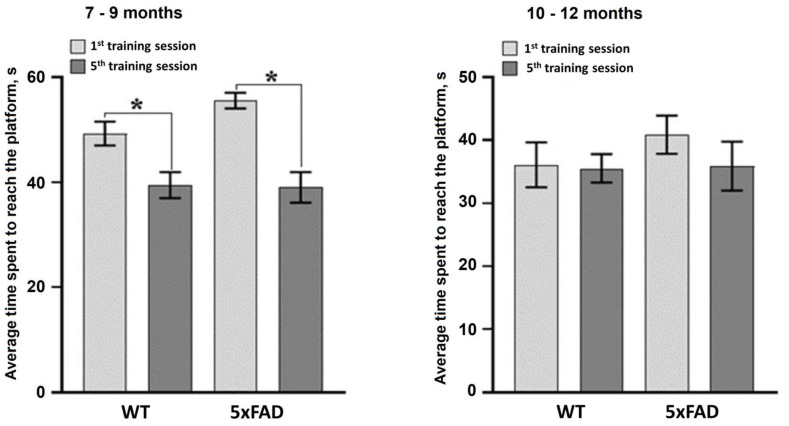
Analysis of learning ability of female WT and 5xFAD mice in the Morris water maze after chronic sleep deprivation modeling. *—versus the first session, *p* < 0.05, the Wilcoxon *t*-test.

Assessment of spatial learning and memory using the Morris water maze test showed that the mice aged 7–9 months retained their learning ability (Figure 1). The average time to reach the platform was significantly reduced in the WT and 5xFAD groups by the 5th training session (Figure 1). In contrast, the mice aged 10–12 months from both the WT and 5xFAD groups were unable to learn and showed no signs of memorizing information (Figure 2). 

**Figure 3:**
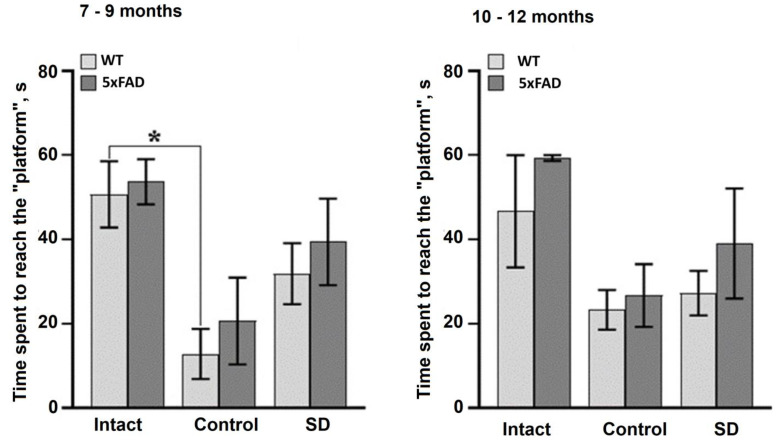
Long-term memory retention test of female WT and 5xFAD mice in the Morris water maze after chronic sleep deprivation modeling. *—versus “Intact”, *p* ≤ 0.05, the Mann–Whitney test.

The data from the delayed test without the platform in the Morris water maze after the chronic sleep deprivation revealed long-term memory impairments in 7–9 months-old WT mice (Figure 3). The time to reach the target in the sleep-deprived WT mice did not differ from that in the untrained (intact) animals, whereas it was significantly reduced in the control mice. There were no significant differences between the trained and untrained 5xFAD mice at 7–9 months of age, or between the WT and 5xFAD mice at 10–12 months of age, demonstrating a lack of a learning and memory reconsolidation processes.

**Figure 4:**
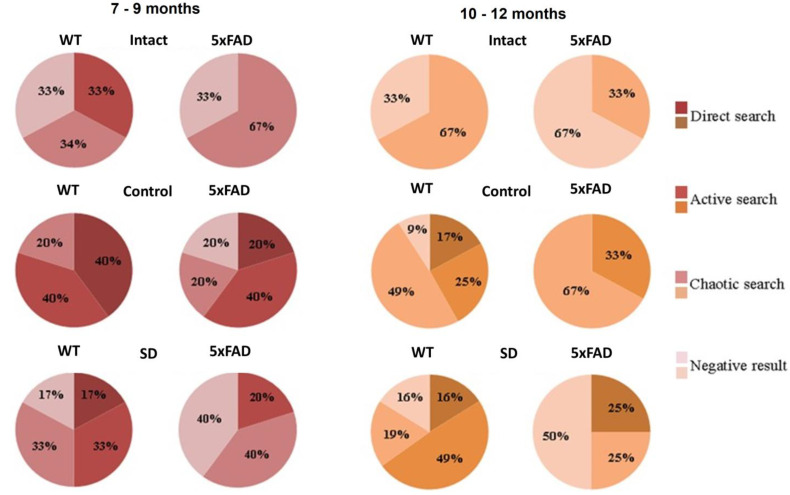
Distributions of target searching strategies of female WT and 5xFAD mice in the long-term memory retention test in the Morris water maze after simulated chronic sleep deprivation.

Evaluation of the platform search strategy after the modeled sleep deprivation revealed a lack of direct search in the 7–9 months-old of 5xFAD mice (Figure 4). In this group, the chaotic strategy or negative attempts to search the platform were predominant, which may indicate the loss of spatial memory traces after the chronic sleep deprivation influence.

Analysis of general locomotor and orienting–exploratory activity of mice in the open field test showed no significant differences between the values for mice aged 7–9 months from both the WT and 5xFAD groups (Supplementary Materials, Table S1A). However, the motor activity of sleep-deprived mice tended to decrease (the number of crossed squares in the periphery of the arena: Control WT 72.5 ± 8.1, Control 5xFAD 62 ± 6.5, SD WT 47.2 ± 2.8, and SD 5xFAD 49.2 ± 11.3; the number of crossed squares in the center of the arena: Control WT 32.5 ± 3.9, Control 5xFAD 29.6 ± 2, SD WT 16.4 ± 4.9, and SD 5xFAD 16.8 ± 3.5). The 5xFAD mice exposed to sleep deprivation also tended to spend more time in the center of the arena and overall showed aggression during the test (Supplementary Materials Table S1A). A decrease in the exploratory activity of mice after sleep deprivation was also noted (the number of upright postures: Control WT 3 ± 0.4, Control 5xFAD 5.6 ± 0.8, SD WT 1.8 ± 0.7, and SD 5xFAD 2.5 ± 0.8).

The 10–12 months-old 5xFAD mice exposed to sleep deprivation demonstrated a pronounced tendency to alter behavioral reactions to a novel environment (Supplementary Materials, Table S1B). In the SD 5xFAD group, the mice spent the shortest time in the arena center (Control WT 67 ± 14.7 s, Control 5xFAD 64 ± 18.3 s, SD WT 72.9 ± 19.4 s, and SD 5xFAD 28.6 ± 5 s) and the increased number of crossed squares on the arena periphery (Control WT 52 ± 8, Control 5xFAD 55.6 ± 7.7, SD WT 59.8 ± 8, and SD 5xFAD 70.3 ± 16.3). The SD 5xFAD mice also showed a trend towards increased exploratory activity (the number of upright postures: Control WT 1.25 ± 0.5, Control 5xFAD 1 ± 1, SD WT 2.9 ± 0.9 and SD 5xFAD 3 ± 2). Overall, the 5xFAD mice from the 10–12 months age group showed signs of stress and anxiety after the modeled sleep deprivation.

Brain cell mitochondrial functional activity assessment revealed no significant differences in the main parameters of the functional state of the brain mitochondrial apparatus of WT and 5xFAD mice from the SD groups compared to the values of the intact and control mice (Supplementary Materials, Table S2). However, it is worth noting that aging increases the risk of a decrease in mitochondrial respiratory chain activity and the intensity of oxidative phosphorylation. All examined parameters of the mitochondrial functional activity in brain cells of the 10–12 months-old mice were reduced compared to the values of the 7–9 months-old age group (Supplementary Materials, Table S2B). The assessment of the oxygen consumption rate by the brain mitochondria showed a downward tendency in mitochondrial respiration activity with inhibition of the respiratory chain complex I and activation of the respiratory chain complex II in mice of the older age group (10–12 months). The decrease in the activity of the first and second complexes of the respiratory chain is considered to be a step towards a general decrease in the animal’s resistance to stress. In the SD WT group, the above parameters were lower than in the SD 5xFAD group (complex I: SD WT 15.3 ± 2.2 pmol/(s·mL), SD 5xFAD 16.6 ± 2.8 pmol/(s·mL); complex II: SD WT 144.3 ± 20.7 pmol/(s·mL), SD 5xFAD 180 ± 18.8 pmol/(s·mL)) (Table S2B). Thus, an assessment of the mitochondrial functional activity revealed an increase in the rates of oxygen consumption, ADP-stimulated respiration, and proton leakage in brain cells of the 5xFAD mice after the influence of sleep deprivation. Such processes in the brain mitochondrial apparatus can be seen as a response to the modeled stress. The activation of mitochondrial functional activity can lead to rapid energy depletion, and, as a result, to cell death.

**Figure 5:**
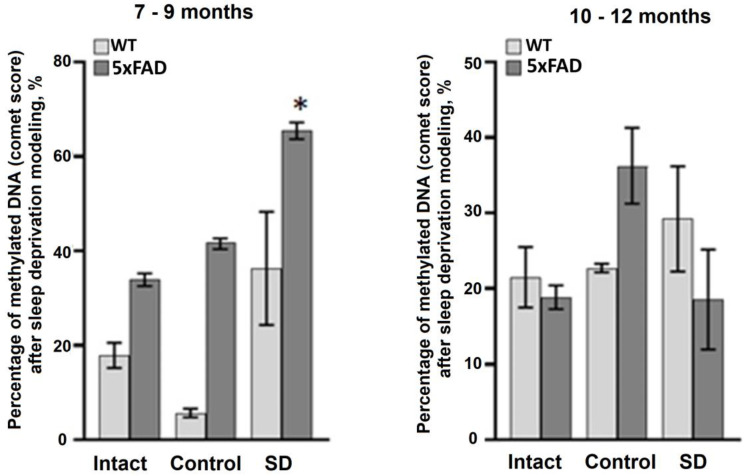
The percentage of methylated DNA (comet score) in the blood samples of female WT and 5xFAD mice after sleep deprivation modeling, *—versus “Intact”, *p* ≤ 0.05, the Mann–Whitney test.

Next, we assessed the level of DNA methylation in blood samples using a method for registration of DNA chain damages (i.e., DNA comet assay). The data obtained revealed an increase in the total level of DNA methylation in the 5xFAD mice aged 7–9 months after the influence of chronic sleep deprivation (Figure 4). Interestingly, the total level of DNA methylation in mice of the 10–12 months-old age groups was lower compared to the 7–9 months-old animals (Figure 5). No significant changes in the DNA methylation rate were registered in the blood samples of both the WT and 5xFAD mice aged 10–12 months.

**Figure 6:**
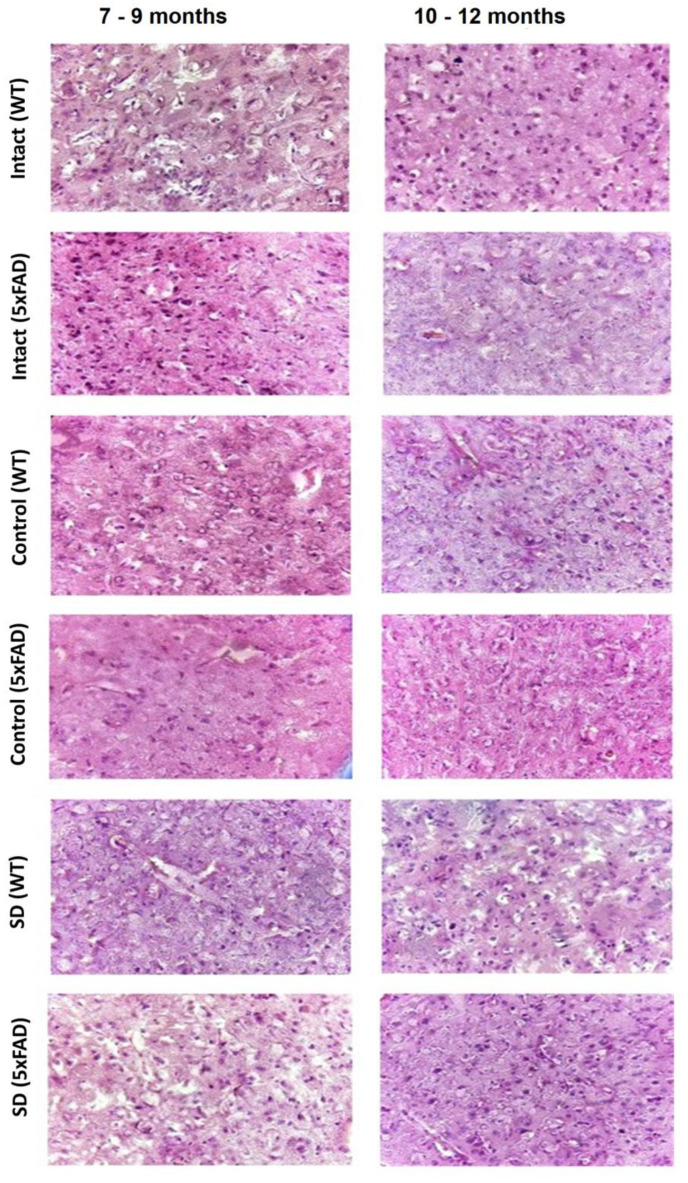
Representative images of histological samples of brain cortex obtained from female WT and 5xFAD mice after sleep deprivation modeling. Hematoxylin-eosin staining, magnification ×20.

Histological analysis revealed no significant morphological changes in the cerebral cortex of 5xFAD female mice in response to learning and memory tests and behavioral assessment (Figure 6). However, the morphometric parameters of the internal granular layer of the cerebral cortex of mice were altered not only in relation to the age but also in relation to the genotype. The cerebral cortex of 7–9-months-old mice from the Intact WT and Intact 5xFAD groups and the 10–12-months-old mice of the Intact WT is represented by large neurons. In the Intact 5xFAD group (10–12 months), there was a decrease in the size of the soma of the nerve cells (Figure 6). The cells are predominantly round or angular (star-shaped). The nuclei have a well-defined structure of a round/oval shape and occupy, on average, half of the cell volume. The cytoplasm lacks large inclusions. 

The cerebral cortex of all control groups of both age groups has a similar morphological structure (Figure 6). However, the cells are 0.1–0.3 times smaller in size. Most cells in 10 fields of view have a high nuclear–cytoplasmic ratio: the nucleus occupies about 1/3 of the total cell volume. 

The influence of sleep deprivation caused pronounced changes in both age groups of WT and 5xFAD mice. In the cerebral cortex of WT mice, a decrease in the branching of the nerve cell processes and the average diameter was observed. The 5xFAD mice showed a decrease in the branching and thickness of neuronal processes, a clear sign of synaptic contact degradation, and a slight increase in the size of nuclei. The increase in the nuclear–cytoplasmic ratio and the weakening of nerve cell outgrowths indicate the aggravation of neurodegenerative processes in the 5xFAD mice of older age group. 

## 4. Discussion

The role of sleep in brain function remains poorly understood. There is evidence to suggest that the release of substances, clearance of intercellular space, and removal of unused synapses during sleep are extremely important components that help the brain to perform its physiological functions [24,25]. However, the existing data do not adequately explain the fundamental role of sleep in the survival of the organism. Numerous observations suggest that sleep deprivation inevitably leads to death [8]. We have previously shown that the adaptive capacity of the mouse brain declines with age [9]. Moreover, sleep deprivation can be considered as a mobilizing and not significantly deleterious stress for young C57BL/6 mice, whereas in adulthood and old age it provokes neurodegeneration, reduces memory retention, and has a negative impact on the animal’s behavior and decreases the adaptive capacity of brain cells, including the increased level of age-related PLIN2 protein expression and a tendency towards decreased DNA methylation levels [9].

Taking into account our previous data, in this study we performed a complex analysis of the influence of sleep deprivation on age-related changes during neurodegenerative processes in an AD model. Clinical evidence suggests that insomnia as a symptom of AD is highly detrimental, predicting rapid hospitalization and death [12,24]. Recent studies suggest that insomnia, especially at a young age, contributes to the early manifestation of the disease and may act as a triggering factor in the development of early-onset AD [3,26]. Our data showed that 5xFAD mice, which serve as a model for the familiar form of AD, were unable to tolerate prolonged sleep deprivation compared to their non-transgenic littermates (WT). Classic models of sleep deprivation in small laboratory animals are based on 11 to 12 h of forced wakefulness [9,17]. Herein, we had to modify the protocol because the 5xFAD mice, aged 10–12 months, had an 80% mortality from 11 h of sleep deprivation. The high mortality rate underlines the extreme importance of sleep for brain function in AD and justifies the need to pay special attention to this syndrome in the clinic.

The use of physiologically valid models both in vitro and in vivo is essential for studying the pathogenesis of neurodegenerative processes. PDAPP mice were the first transgenic line carrying the Indian mutation of human amyloid precursor protein APP (V717F) [27]. These mice already produce pathological amyloid beta 1-42 (Aβ1-42) at an early postnatal period. PDAPP mice exhibit the main pathological manifestations of AD, including accumulation of extracellular amyloid plaques, hyperphosphorylation of tau protein, and synaptic dysfunction. On the other hand, no accumulation of neurofibrillary tangles and intracellular amyloid as well as the development of neurodegeneration was observed [27]. Therefore, another transgenic Tg2576 mouse line carrying the Swedish mutation of human APP (K670N/M671L) was created. In this line, the amyloid plaques were detected at 11–13 months of age, but no neurodegenerative processes were observed in the hippocampus [28]. The 3xTg-AD mice were the first line with sustainable all-phenotypic manifestations of AD [29]. In addition to APP (K670N/M671L) and PS1 (M146V) mutations, the 3xTg-AD mice carry a tau-protein mutation Tau (P301L) [29]. 

To date, the most widely used line is the 5xFAD mouse line, which carries the mutations in human genes associated with the familial from of AD, including five mutations in the APP and presenilin1 (PSEN1) human transgenes (APP KM670/671NL (the Swedish mutation), APP I716V (the Florida mutation), APP V717I (the London mutation), and PSEN1 (M146L, PSEN1 L286V) which contribute to the rapid development of amyloidosis. Proteolysis of APP leads to the formation of amyloid beta, a major component of amyloid plaques. PSEN1 is a subunit of γ-secretase and is associated with either an increase in total Aβ levels or an increased ratio of its toxic variant Aβ42 compared to Aβ40 [30], leading to amyloid plaque formation and AD progression [31]. Thus, the 5xFAD mouse is one of the best experimental models for studying the familial form of AD and is suitable for studying the theory of the amyloid cascade as the cause of AD, since all the signs, such as accumulation of extra- and intracellular Aβ, disruption of synaptic transmission, and neurodegeneration are phenotypically manifested [32].

The first signs of memory impairment in the 5xFAD mice begin at 4 months of age. The classical form of AD, associated with the accumulation of amyloid beta aggregates, is observed in 5xFAD mice by the 8th month of life [33]. In this study, the following two life stages of the 5xFAD mice were analyzed: 7–9 months, the period of clinical manifestation of the modeled AD, and the period of 10–12 months, which is considered a stage of old age for this line.

The current study showed that long-term memory formation is impaired in 5xFAD mice at 7–9 months of age, which is characteristic of AD progression and confirms the relevance of this animal model. The data from the delayed test in the Morris water maze revealed that the time spent to reach the target in 5xFAD mice 7–9 months old did not differ from the values of the untrained animals, whereas in wild-type mice it was significantly lower than the trained animals. By 10–12 months of age, memory disturbances were evident in both the wild-type and 5xFAD mice.

Modeling 9 h of sleep deprivation caused unidirectional changes in 5xFAD mice of different age groups associated with a deterioration in spatial memory reconsolidation and an increase in neurodegenerative processes, including the morphological changes in the cerebral cortex. The changes observed are more pronounced in the older group of animals, indicating the extremely low adaptive capacity of their organism.

Of particular interest are the data assessing the total level of DNA methylation in blood samples from 5xFAD mice. In mammals, age-related hypomethylation processes associated with a decrease in DNA methylase activity have been observed and can be considered as one of the main mechanisms of aging [34,35]. Total DNA hypomethylation is accompanied by hypermethylation of individual CpG sites and changes in the activity of certain genes [34,36]. Age-related acceleration, which is characteristic of conditions such as Down syndrome, HIV infection, and AD, is associated with an increased rate of epigenetic changes [37,38,39,40]. Increased levels of DNA methylation in the blood were found in sleep-deprived 5xFAD mice aged 7–9 months. In contrast, the stress-induced increase in total DNA methylation was not detected in the blood of 5xFAD mice from the older group (10–12 months). The data obtained are of particular interest because, on the one hand, aging is accompanied by a total hypomethylation of DNA as a result of a decrease in the activity of DNA methylases [41], while, on the other hand, it is shown that the activity of DNA methylase type 1 (DNMT1), which provides supporting methylation, and, consequently, the total methylation of DNA, can increase under stress [42]. It could be assumed that the adaptive response to sleep deprivation in 5xFAD mice involves activation of methylation processes, whereas in old mice the lack of effect is associated with a decrease in age-mediated dysregulation of DNMT1 activity. This could be an interesting direction for further research.

In spite of the valuable results obtained, which extend our knowledge of the contribution of chronic sleep deprivation to age-related neurodegenerative processes in Alzheimer’s disease, the study has some limitations. The 5xFAD mice represent a relevant model of familial AD; however, this form of the disease represents only 5–10% of the total number of cases [43]. Therefore, further research on other AD models will help to verify and strengthen our results. Another limitation is the use of only female mice as study objects. A comparative analysis of the effects of sleep deprivation on male and female 5xFAD mice and the identification of sex-specific effects of insomnia on the development of neurodegenerative progression in AD is an intriguing direction for further research. 

## 5. Conclusions

Chronic sleep deprivation is a limiting factor for female 5xFAD transgenic mice possessing a familial form of AD. The 5xFAD mice cannot tolerate chronic sleep deprivation for more than 9 h. The mortality of the mice increases sharply starting from 2 to 3 days of the experiment. Chronic sleep deprivation was shown to affect spatial memory reconsolidation processes in mice with a familial form of Alzheimer’s disease. The female 5xFAD mice demonstrated pronounced stress reactions, including aggressive behavior. There was a degradation of neuronal processes in the cerebral cortex. Chronic sleep deprivation results in an increase in the total level of DNA methylation, indicating the deep molecular mechanisms of body adaptation in mice.

## Figures and Tables

**Table 1:** Distribution of WT and 5xFAD mice into experimental groups.

**Experimental Group/Number of Mice per Group**		**Intact**	**Control**	**SD**
		The Mice Were Not Trained in the Morris Water Maze, but Participated in the Delayed Test (without the Platform) as a Baseline	The mice not Exposed to Chronic Sleep Deprivation	The Mice Exposed to Chronic Sleep Deprivation
7–9 months	WT	10	10	15
	5xFAD	10	10	15
10–12 months	WT	10	10	15
	5xFAD	10	10	15

**Table 2:** Weight difference of female WT and 5xFAD mice between the beginning and the end of chronic sleep deprivation modeling.

Experimental Group		Intact	Control	SD
7–9 months	WT	0.4 ± 0.7	0.4 ± 1.6	−0.13 ± 0.4
	5xFAD	1.3 ± 0.2	1.3 ± 0.5	0.7 ± 0.6
10–12 months	WT	0.4 ± 0.3	0.01 ± 0.3	−0.6 ± 0.5
	5xFAD	1.2 ± 0.6	1.2 ± 0.3	4.6 ± 2.5 ^*

The values are presented in grams. ^ versus “SD”, * versus age-matched WT group, *p* ≤ 0.05, ANOVA test.

## Data Availability

The data used to support the findings of this study are available from the corresponding author upon request.

## Supplementary Materials

The following supporting information can be downloaded at: https://www.mdpi.com/article/10.3390/neurolint15030049/s1, Table S1: Parameters of behavioral reactions of female WT and 5xFAD mice in the Open field test day after sleep deprivation modeling; Table S2: Main parameters of the brain mitochondrial functional activity of female WT and 5xFAD mice after sleep deprivation modeling, pmol/(s·mL).
