# Supplementary material for: Contribution of Chronic Sleep Deprivation to Age-Related Neurodegeneration in a Mouse Model of Familial Alzheimer’s Disease (5xFAD)
Source: Neurol Int. 2023 Jun 27;15(3):778–91. doi: 10.3390/neurolint15030049 (PMC10366916; doi:10.3390/neurolint15030049)
Supplement: Supplementary file 1 [file neurolint-15-00049-s001.zip › neurolint-2427136-supplementary.pdf]

Table S1. Parameters of behavioral reactions of female WT and 5xFAD mice in the Open field test day after sleep deprivation modeling

A. 7-9 months

Experimental group	Acts of defecation	Acts of urination	Grooming, s		Number of upright postures	Number of squares passed in the arena		Time spent in the center of the arena, s	Number of peeks in holes
			Short	Long		Periphery	Centre		
Intact WT	0±0	0.6±0.3	4±2.1	1±1	3±1.5	30.3±21.1	17.3±13.9	129.6±87.1	21.6±8.8
Intact 5xFAD	0±0	2±0.7	3±1.7	1.6±0.8	0.6±0.6	61±7.9	33.3±4.9	99.6±3.4	17.3±6.6
Control WT	0.5±0.3	0.75±0.75	2.5±0.9	1±0.4	3±0.4	72.5±8.1	32.5±3.9	70.7±13.4	32±2.5
Control 5xFAD	0±0	0.6±0.3	6.6±4.8	3.3±2.4	**5.6±0.8***	62±6.5	29.6±2	68±4	18±2.3
SD WT	0±0	0.4±0.2	2.2±2.2	0.4±0.2	1.8±0.7	47.2±2.8	16.4±4.9	88.4±21.1	34±4.4
SD 5xFAD	0±0	1±0.4	9.3±2.4	2.8±1.2	2.5±0.8	49.2±11.3	16.8±3.5	139.3±26.5	16.6±4.6

B. ≤12 months

Experimental group	Acts of defecation	Acts of urination	Grooming, s		Number of upright postures	Number of squares passed in the arena		Time spent in the center of the arena, s	Number of peeks in holes
			Short	Long		Periphery	Centre		
Intact WT	0±3	1.6±0.3	3.6±2.7	0.3±0.3	0.3±0.3	19.3±8.7	14.3±3.8	139.3±63.6	14.6±3.5
Intact 5xFAD	0±0	2.3±0.8	1.6±1.6	0.3±0.3	2.3±0.6	53.6±9.6	21.6±5.7	62.6±21.6	16.6±5.8
Control WT	0.1±0.1	1±0,5	4.9±2.4	1±0.4	1.25±0.5	52±8	18.9±4.4	67±14.7	23.1±3.9
Control 5xFAD	0±0	1.3±0.3	4.3±2.9	1.3±1.3	1±1	55.6±7.7	15±2.5	64±18.3	22.6±3.7
SD WT	0±0	1.9±0.4	5±1.6	1.2±0.6	2.9±0.9	59.8±8	20.5±5.6	72.9±19.4	24.9±4.8
SD 5xFAD	0±0	0.6±0.3	2±2	0.6±0.6	3±2	70.3±16.3	14.6±2.6	28.6±5	25.6±5.2

* - versus “Intact”, ANOVA test, p≤0.05

Table S2. Main parameters of the brain mitochondrial functional activity of female WT and 5xFAD mice after sleep deprivation modeling, pmol/(s*mL)

A. 7-9 months

Experimental group	Basal oxygen consumption rate (V4)	ADP-stimulated respiration (V3)	Inhibition of NADH dehydrogenase (inhibition of respiratory chain complex I)	Activation of an alternate pathway for the respiratory chain (activation of respiratory chain complex II)	Proton leakage
Intact WT	51.6±4.4	228.3±17.9	13.6±3.3	192.3±14.5	58.6±3.4
Intact 5xFAD	52±3.6	241±11.5	19.6±1.8	214.3±10.7	66±3.8
Control WT	70.6±14.8	242.2±43.1	25.8±3.7	199.2±24.2	98.6±18.2
Control 5xFAD	65.8±17.2	210.8±29.8	23.2±4.2	193±24.1	91.6±21.5
SD WT	70.3±10	217.8±9.7	20.8±3.1	182.2±8.3	98.6±8.5
SD 5xFAD	78.8±15.9	227.8±18.4	23.6±3.7	208.6±16.3	104.8±16.8

B. ≤12 months

Experimental group	Basal oxygen consumption rate (V4)	ADP-stimulated respiration (V3)	Inhibition of NADH dehydrogenase (inhibition of respiratory chain complex I)	Activation of an alternate pathway for the respiratory chain (activation of respiratory chain complex II)	Proton leakage
Intact WT	44.6±4.8	83±4.4	11.3±2	100±17.7	43±6.5
Intact 5xFAD	68±20.4	160±48.9	14.3±2.4	149.3±45.2	72.6±27.1
Control WT	80.3±12.4	205.3±32.4	17.1±3.7	185±30.7	97.8±19.2
Control 5xFAD	54±2.5	179.6±7.3	9±1	116±6	45±4.9
SD WT	68.8±8.6	154.3±24.3	15.3±2.2	144.3±20.7	80.1±11.3
SD 5xFAD	67.7±5.3	225±25.3	16.6±2.8	180±18.8	76.7±6.4

No statistical differences between groups, $p>0.05$, ANOVA test
